# Diabetes Mellitus and Vitamin D Deficiency: Comparable Effect on Survival and a Deadly Association after a Myocardial Infarction

**DOI:** 10.3390/jcm9072127

**Published:** 2020-07-06

**Authors:** Aneta Aleksova, Federico Ferro, Giulia Gagno, Laura Padoan, Riccardo Saro, Daniela Santon, Elisabetta Stenner, Giulia Barbati, Chiara Cappelletto, Maddalena Rossi, Antonio Paolo Beltrami, Gianfranco Sinagra

**Affiliations:** 1 Cardiovascular Department, Azienda Sanitaria Universitaria Giuliano Isontina (ASUGI) and Department of Medical Surgical and Health Sciences, University of Trieste, 34100 Trieste, Italy; fferro@units.it (F.F.); GIULIA.GAGNO@studenti.units.it (G.G.); riccardo.saro@gmail.com (R.S.); chiaracappelletto91@gmail.com (C.C.); maddalenarossi93@gmail.com (M.R.); gianfranco.sinagra@asugi.sanita.fvg.it (G.S.); 2 Azienda Ospedaliera di Perugia and University of Perugia, Cardiology and Cardiovascular Physiopathology, 06156 Perugia, Italy; argonauta92@hotmail.it; 3 Cardiovascular Department, Azienda Sanitaria Universitaria Giuliano Isontina (ASUGI), 34100 Trieste, Italy; daniela.santon@asugi.sanita.fvg.it (D.S.); elisabetta.stenner@asugi.sanita.fvg.it (E.S.); 4 Biostatistics Unit, Department of Medical Surgical and Health Sciences, University of Trieste, 34100 Trieste, Italy; gbarbati@units.it; 5 Department of Medicine, University of Udine, 33100 Udine, Italy; antonio.beltrami@uniud.it

**Keywords:** diabetes mellitus, vitamin D, hypovitaminosis D, mortality, heart failure, myocardial infarction, angina, prognosis, MACE, RAAS

## Abstract

Survivors after a myocardial infarction (MI), especially those with diabetes mellitus (DM), remain at high risk of further events. Identifying and treating factors that may influence survival may open new therapeutic strategies. We assessed the impact on prognosis of DM and hypovitaminosis D (hypovitD), alone or combined. In this prospective, observational study, 1081 patients were enrolled surviving an MI and divided into four groups according to their diabetic and VitD status. The primary end-point was composite of all-cause mortality, angina/MI and heart failure (HF). Secondary outcomes were mortality, HF and angina/MI. During a follow-up of 26.1 months (IQR 6.6–64.5), 391 subjects experienced the primary end-point. Patients with DM or hypovitD had similar rate of the composite end-point. Patients with only hypovitD or DM did not differ regarding components of composite end-point (angina *p* = 0.97, HF *p* = 0.29, mortality *p* = 0.62). DM and VitD deficiency had similarly adjusted risks for primary end-point (HR 1.3, 95%CI 1.05–1.61; HR 1.3, 95% CI 1.04–1.64). The adjusted HR for primary composite end-point for patients with hypovitD and DM was 1.69 (95%CI 1.25–2.29, *p* = 0.001) in comparison to patients with neither hypoD nor DM. In conclusion, DM and hypovitD, individually and synergistically, are associated with a worse outcome after MI.

## 1. Introduction

Cardiovascular diseases and diabetes mellitus (DM) are two important burdens on health worldwide and their prevalence is predicted to continue to rise if current trends prevail [1]. Besides the deleterious effects of DM, its vascular complications [2] decrease patients’ life expectancy and quality of life. Although mortality caused by myocardial infarction (MI) is declining, survivors after MI, especially those with DM, remain at high risk of further cardiovascular events [3].

Many recurrent cardiovascular events could be prevented or delayed by applying interventions to better control cardiovascular risk factors, particularly in patients with DM [4].

Vitamin D (VitD) deficiency, a worldwide health problem, emerged in the last years as a cardiovascular risk factor, being associated with the development of coronary atherosclerosis [5] and with a worse prognosis of patients surviving MI [6,7].

VitD deficiency is common in DM [8] and diabetic patients with complications have lower levels of serum VitD, compared to those without any [9,10]. In patients with MI, VitD deficiency is associated with poorly controlled glucose homeostasis through increased insulin resistance and decreased insulin secretion [11]. Also, VitD reduces renin–angiotensin–aldosterone system (RAAS) activity through downregulation of renin secretion [12]. Furthermore, hypovitaminosis D (hypovitD) correlates with subsequent adverse cardiac remodeling and mortality [6,13].

Therefore, the evaluation of additional hazards associated with insufficient VitD levels and DM among patients surviving MI could provide important indication for the clinical practice management.

In this study, we assessed the prognostic impact of DM and insufficient levels of VitD, alone or in combination, in patients with previous MI.

## 2. Materials and Methods

### 2.1. Study Population

In this observational, prospective study, we enrolled 1081 adult patients, surviving an acute MI. All patients underwent urgent coronary catheterization, transthoracic echocardiogram and were subjected to routine blood tests. VitD was measured from fresh plasma samples. A chemiluminescent test (CLIA) was performed using Liaison (DiaSorin Inc, Saluggia, Italy) instrument.

### 2.2. Study Design

We divided our population into four groups according to DM and VitD status: group 1 included patients with DM and hypovitD, group 2 patients with only hypovitD, group 3 patients with only DM and group 4 patients without DM nor hypovitD.

### 2.3. Definitions and End-points

HypovitD was defined as serum levels of 25-hydroxy vitamin D (25(OH)D3) ≤ 20 ng/mL [14,15]. Diabetic status was assessed during the index hospitalization for MI. Patients with history of diabetes managed with diet, insulin or oral medications or with HbA1c level ≥ 6.5% during the index hospitalization were considered diabetics.

The primary end-point was time to first major event, composite of all-cause mortality, angina/MI or HF during follow-up. Secondary outcomes were mortality, HF and angina/MI during follow-up. Angina/MI were diagnosed according to ESC guidelines [16,17]. Multi-vessel critic coronary artery disease was considered as the presence of > 70% stenosis in at least two coronary vessels at angiography. Multistage revascularization was not considered an end-point. HF was diagnosed in presence of shortness of breath, fatigue, leg edema at rest or on exertion and necessity of diuretic prescription or an increase in the daily dosage of the ongoing treatment or hospitalization for HF [18].

All patients were followed from hospital discharge to the end of follow-up (date of death or last contact with the patient). The median duration of follow-up was 26.1 months (IQR 6.6–64 months). Information regarding end-points were achieved from the hospital database Cardionet (INSIEL, Trieste, Italy). This study was performed in accordance with the declaration of Helsinki and was approved by the Institutional Regional and Hospital Ethics Committee (N 67/2015, update CEUR-2019-Em-44 dd. 12/02/2019 PROT 4214/P/GEN/ARCS).

### 2.4. Statistical Analysis

Continuous variables are shown as mean ± standard deviation or median (interquartile range), as suitable. Patients’ characteristics across groups are compared using the Student t-test or the Mann–Whitney test as applicable. Categorical variables are presented as percentages and are compared using the Chi-square test or the Fisher exact test if needed.

By using the Kaplan–Meier method, the cumulative probability of primary composite end-points was estimated during follow-up. Afterwards, the cumulative incidence curves of the secondary end-points of angina/reinfarction or HF were estimated and compared across groups considering death as a competing risk, using the R library “cmprsk” that implements the method described by Gray et al. [19]. Multivariable Cox proportional hazards models were used to evaluate the relationship between patients’ data and primary and secondary outcomes (in the latter case using cause-specific models). In the multivariable models, variables significant at the univariable Cox analysis and those clinically relevant for the specific event were retained. Subsequently, initial multivariable models were reduced by means of a backward conditional stepwise procedure in order to minimize collinearity among predictors. Plots of the smoothed estimates of covariates versus the probability of event were used to check the linearity assumption. The proportional hazard assumption for covariates selected in the multivariable model was checked by means of the Therneau test. A nomogram derived from the multivariable model has been estimated [20]. A two-tailed *p* < 0.05 was considered statistically significant for all test results. All analyses were performed using the software IBM SPSS Statistical Package for Windows, version 19 and the R statistical software.

## 3. Results

### 3.1. Patients Characteristics

We enrolled 1081 patients surviving an acute MI. Baseline variable for the whole cohort and for groups are presented in Table 1.

Most enrolled patients were male, with mean age of 66.7 ± 11.5 years. One third of the population (361 patients; 33.4%) were diabetic: 315 patients (29.1%) were diagnosed with DM before admission, while in 46 patients (4.2%) the diagnosis of DM was made during the index hospitalization.

The median level of VitD was 16.7 (9.4–23.7) ng/mL. HypovitD was highly prevalent (63%) and was more frequent among patients with previously known (70.2%) and newly diagnosed DM (73.9%) when compared to non-diabetic patients (59.2%). Gender distribution (male gender 83% vs. 72.9%, *p* = 0.041, respectively) and history of previous cardiovascular events (22 vs. 32.1%, *p* = 0.043, respectively) were significantly different between diabetic patients with and without hypovitD.

In order to analyze the impact of DM and VitD deficiency on outcome, we divided our population into four groups: group 1 comprised 255 patients (23.59%) with DM and hypovitD, group 2 comprised 426 patients (39.41%) with only hypovitD, group 3 comprised 106 patients (9.8%) with only DM and group 4 comprised 294 patients (27.2%) without DM and hypovitD.

Compared with patients with only DM (group 3), those presenting only hypovitD (group 2) were more frequently female, had higher cholesterol levels and BMI. Patients with DM only, compared with patients with hypovitD, more frequently had cardiovascular risk factors and previous cardiovascular events. At predischarge echocardiographic evaluation, there was no difference between groups 2 and 3 in the left ventricular’s (LV) dimensions and function, wall motion score index and mitral insufficiency. Also, there was no difference between these two groups regarding type of MI, timing, percentage and revascularization strategy, renal function and treatment medication during follow-up.

In comparison to the other three groups, patients from group 1 (both DM and hypovitD) tended to be admitted with a worse clinical presentation (Killip > 2), with a multivessel disease and underwent more frequently surgical revascularization (*p* < 0.001).

### 3.2. Clinical Outcome

**Figure 1:**
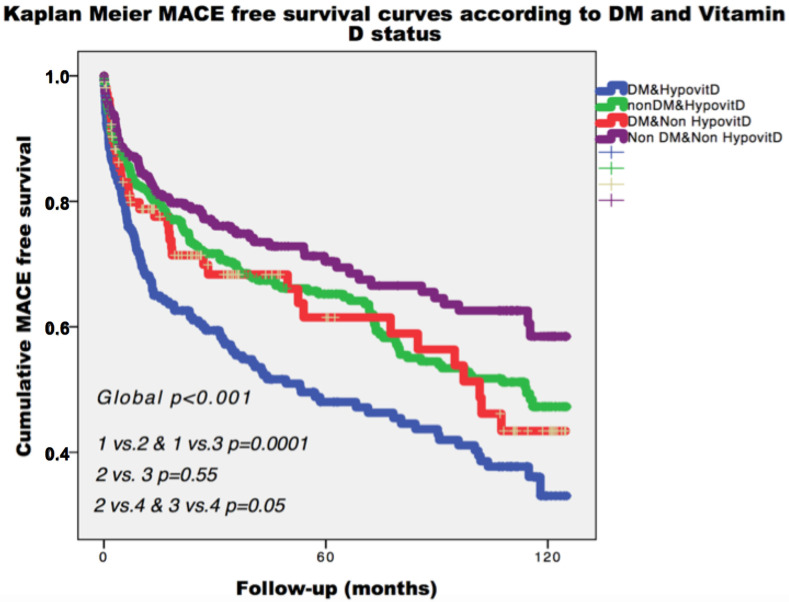
Kaplan–Meier curves for primary end-point, survival according to diabetes and Vitamin D status. Legend: MACE: major adverse cardiac events; DM: diabetes mellitus; HypovitD: hypovitaminosis D.

During a median follow-up of 26.1 (6.6–64.5) months, the composite end-point occurred in 391 patients (36.2%). As depicted in Figure 1, patients from group 4 (no DM, nor hypovitD) had the most favorable prognosis during follow-up. Kaplan–Meier analysis showed that patients with DM or VitD deficiency had similar rate of the composite end-point (44.9% vs. 40.7%, *p* = 0.55, Figure 1). Among diabetic patients, the composite end-point rate during follow-up increased in the presence of hypovitD (48.6%, Figure 1).

Further, to estimate the cumulative incidence of angina/MI and HF with the competing risk of death, competing risk analyses were conducted. These analyses showed that, while 8% of patients experienced HF as a first event at 24 months of follow-up, 10% of patients died within the same timeframe. At 96 months, these cumulative incidences of events rose to 14% for HF and 22% for death. Concerning angina/MI as a first event, 13% of patients experienced it at 24 months of follow up. At 96 months, the cumulative incidence of angina/MI rose to 19%.

Cumulative incidences for cause-specific end-points at different follow-up time points across groups are shown in Tables S1 and S2. No significant differences across groups were observed for the specific risk of angina/MI (Figure S1). Over the entire follow-up, patients with hypovitD and DM had a risk of HF and death about two times greater compared with patients without VitD deficiency and DM (*p* < 0.001 for both events) (Figures S2 and S3).

In pairwise comparisons, patients with only VitD deficiency or DM did not differ regarding components of composite end-point (for angina *p* = 0.97, for HF *p* = 0.29, for mortality *p* = 0.62), (Figures S1–S3). Patients with only hypovitD had significantly lower cumulative mortality and HF incidence compared with patients presenting both insufficient VitD levels and DM (*p* = 0.007 and *p* < 0.001, respectively) (Figures S2 and S3).

At univariable analysis, VitD deficiency was associated with an increased risk of major events (HR 1.42, 95% CI 1.14–1.77, *p* = 0.002). Diabetic patients had also increased risk of major cardiovascular events (HR 1.57, 95% CI 1.28–1.91, *p* < 0001). Considering only diabetic patients of our population, we observed that those with concomitant VitD deficiency had higher hazard of events during follow-up (HR 1.4, 95% CI 1–1.94, *p* = 0.049) as compared to those with isolated DM.

In the adjusted Cox model including age, gender, season, presence of multivessel disease, previous coronary events/revascularization, C-reactive protein (CRP), glomerular filtration rate, LV ejection fraction, treatment with ACE-inhibitors/ARBs and beta blockers, both DM (HR 1.3, 95% CI 1.05–1.61, *p* = 0.014) and VitD deficiency (HR 1.3, 95% CI 1.04–1.64, *p* = 0.022) remained independently associated with the composite end-point (Table 2).

Afterwards, to further analyze the combined effect of VitD deficiency and DM, a four-level variable was inserted into the model; the adjusted hazard ratio for primary composite end-point for patients with VitD deficiency and DM was 1.69 (95%CI 1.25–2.29, *p* = 0.001) in comparison to patients with neither VitD deficiency nor DM.

**Figure 2:**
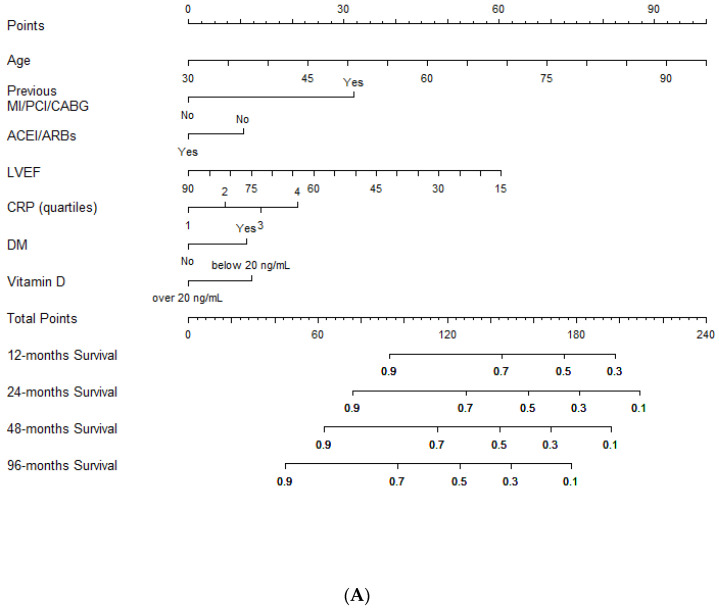

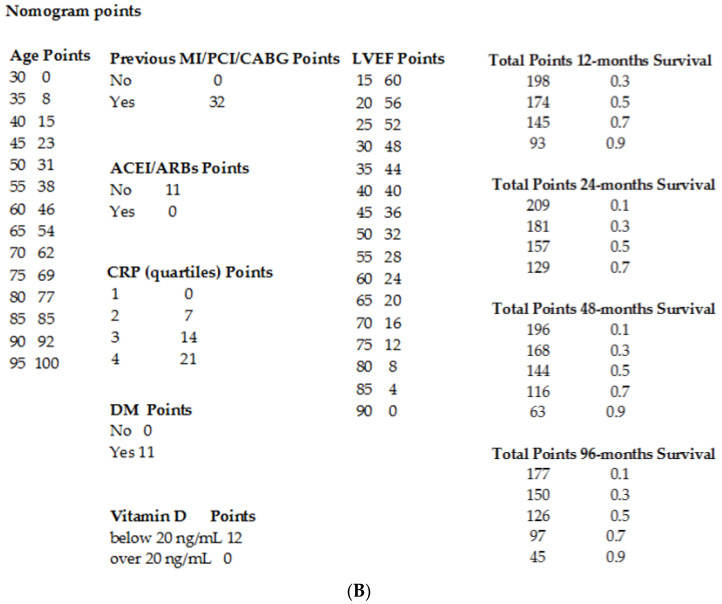
Outcome prediction. (**A**). Nomogram for outcome prediction. (**B**). Clinical individual variables values (derived from the nomogram) and relative score used to predict the likelihood of the composite end-point risk at 12, 24, 48 and 96 months. Legend: MI: myocardial infarction; PCI: percutaneous coronary intervention; CABG: coronary artery bypass grafting; LVEF: left ventricular ejection fraction; CRP: C-reactive protein; ACEIs/ARBs: angiotensin converting enzyme inhibitors/angiotensin receptor blockers; DM: diabetes mellitus.

Lastly, a nomogram for an easy estimation of the risk of the composite end-point was plotted based on the multivariable Cox regression results (Figure 2A,B).

Clinicians should simply draw a perpendicular line from variables to the point line and then sum individual variable scores in the total points line. Lastly, a vertical line should be plotted from the total point to the probability line to determine the likelihood of 12, 24, 48 and 96 months of the composite end-point risk (Figure 2A).

## 4. Discussion

In this cohort study, we reported for the first time that DM and low VitD levels had a similar impact on outcome after acute MI. Remarkably, DM and VitD retained their independent predictive value even after adjusting for other important predictors of outcome. Furthermore, we observed that the combination of DM and insufficient VitD levels exerted powerful negative effect on survival.

VitD deficiency [21] and DM [22] are very common worldwide. HypovitD is an independent risk factor for coronary artery disease (CAD) [5,23] and is associated with adverse outcomes [6,7]. DM is also related with higher risk of CAD and cardiovascular mortality [3]. Additionally, the prevalence of CAD is more than double among diabetic patients with low VitD levels when compared with diabetics with normal VitD levels [24]. Many studies have found direct and indirect correlations between VitD deficiency, insulin secretion and type I [25,26,27,28] or type II DM [29,30]. Furthermore, VitD impacts insulin sensitivity by modulating calbindin and calcium concentration [31], parathyroid hormone [32] and peroxisome proliferator-activated receptor delta production [33] or β-cells inflammation-induced apoptosis [34].

**Figure 3:**
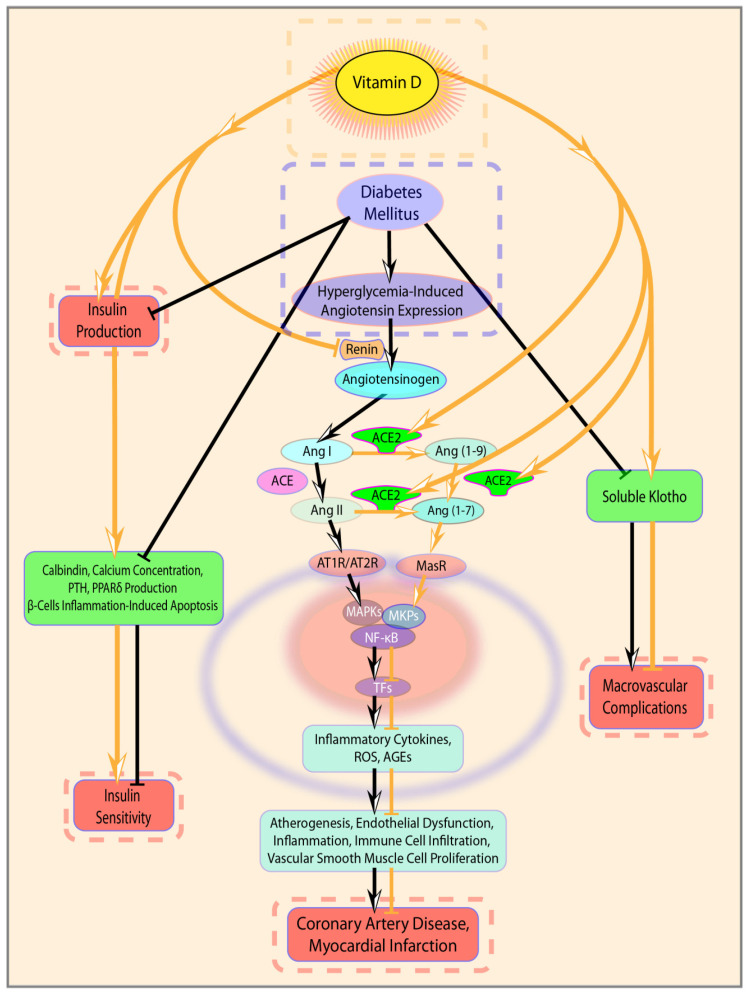
Schematic highlighting of the potential interaction between VitD and DM and the resulting negative effects in coronary artery disease (CAD) pathogenesis and progression. Legend: Angiotensin converting enzyme (ACE); Angiotensin converting enzyme 2 (ACE2); Angiotensin I (Ang I); angiotensin II (Ang II); angiotensin 1–7 (Ang 1–7); Ang II type 1 receptor (AT1R); Ang II type 2 receptor (AT2R); MAS receptor 1 (MAS1); mitogen-activated protein kinase phosphatase (MKPs); mitogen-activated protein kinases (MAPKs); nuclear factor kappa-light-chain-enhancer of activated B cells (NF-kB); transcription factors (TFs); Reactive oxygen species (ROS); advanced glycated end products (AGEs).

VitD deficiency and DM intimately cooperate in different pathogenic mechanisms of coronary atherogenesis and progression (Figure 3), such as endothelial dysfunction, increased pro-inflammatory cytokines production, immune cell infiltration and vascular smooth muscle cell proliferation. Specifically, high glucose levels prompt a cascade of processes, among which are generation and production of proinflammatory cytokines, products of oxidative stress and advanced glycated end-products (AGEs) [33]. Furthermore, elevated glucose levels stimulate RAAS, so hyperglycemia, through enhanced renin activity, stimulates local angiotensin (Ang)II synthesis [34]. Angiotensin II is an activator of nuclear factor kappa-light-chain-enhancer of activated B cells (NF-κB) inflammatory pathways and exerts proatherogenic, pro-inflammatory and pro-oxidant effects [35]. In fact, activation of NF-κB is implicated in pathological inflammation, promoting the transcription of pro-inflammatory cytokines, such as TNF-α and IL-6, which are involved in the progression of coronary atherosclerosis [36].

On the other side, VitD acts at different levels of cardiovascular homeostasis, controlling cell proliferation, regulating DNA repair, exerting anti-inflammatory and anti-oxidative effects and inhibiting RAAS [37,38,39,40]. VitD is effective in reducing the expression of pro-inflammatory cytokines and increasing the expression of those with anti-inflammatory activity. In fact, VitD administration was demonstrated to reduce Th1 cytokines expression (e.g., TNF-α, interferon-γ), but not Th2 cytokines, and to increase the expression of anti-inflammatory cytokines, upregulating mitogen-activated protein kinase phosphatase 1 and suppressing P38 mitogen-activated protein kinases activation [41,42]. More specifically, VitD signaling negatively regulates the NF-κB pathway [40,41,42,43], decreasing the expression and production of pro-inflammatory cytokines including IL-1β, TNF-α, and IL-6 [34]. Moreover, by blocking NF-κB signaling pathway, VitD attenuates hyperglycemia-induced angiotensin expression [44]. VitD effects on RAAS could be explained in light of its downregulation on renin gene expression and its consequent up-regulative influence on the angiotensin converting enzyme-2 ACE2/Ang(1–7)/MasR pathway [12], which is known to have a protective role in the context of CAD and MI [12,45,46]. In addition, the same VitD/ACE2/Ang(1–7)/MasR axis has been confirmed to be involved in the reduction of Ang II-induced ROS accumulation, thus also demonstrating VitD anti-oxidant action [12]. Noteworthy, VitD also has a direct effect on ACE and ACE2, down-regulating ACE and up-regulating ACE2 expression both in vivo and in vitro in high glucose conditions [46,47,48].

Finally, VitD can exert its beneficial effects via Klotho, one of its target genes that has been associated with longevity and whose protective effects against macrovascular complications of type 2 Diabetes were recently described [49]. Klotho is a transmembrane protein mainly expressed in the kidneys, whose extracellular domain can be cleaved to generate a soluble Klotho ectodomain [50]. Membrane-bound Klotho acts as a coreceptor required for fibroblast growth factor (FGF) 23 signaling [51]. The latter is a phosphaturic hormone regulating inorganic phosphate serum levels, as well as parathyroid hormone and 1.25-dihydroxyvitamin D_3_ production [52]. Soluble Klotho can both: a) bind to FGF23/FGF receptor [53] and may prevent vasoconstriction induced by phosphate and FGF23 by increasing nitric oxide production in endothelial cells [54]; and b) exert other, complex, FGF23-independent, hormone-like cardioprotective functions [51]. Importantly, chronic kidney disease, which eventually progresses to cardiovascular disease, is characterized by reduced Klotho levels and FGF23 up-regulation [55]. Similarly, mouse models lacking Klotho and overexpressing FGF23 are characterized by vascular calcification [51]. Conversely, excessively high levels of circulating Klotho are associated with hypophosphatemia and hypocalcemia, increased FGF23 production, bone rarefaction, osteomalacia and fractures [55,56,57].

In line with previously mentioned evidence, in our study survivors of MI with DM and concomitant VitD deficiency had a worse prognosis when compared to those with only DM, hypovitD or neither of them. Samefors et al. [58], in a cohort of 698 patients with type 2 DM, found that low 25(OH)D3 is associated with an increased cardiovascular morbidity/mortality. In our population, diabetic patients had worse long-term outcomes when compared to non-diabetic patients, coherently with previously published data [59].

Surprisingly, we did not observe any difference in survival among patients with only DM or VitD deficiency. Moreover, both were independent predictors of long-term outcomes after adjustment for other covariates, including age, gender, previous cardiovascular events, severity of CAD, treatment, LV and renal function. This fact highlights the importance of an early diagnosis of both these conditions. In addition to strategies for better glycemic control, it is important to apply interventions to slow the progression of CAD and other post-infarct adverse events as soon as possible, searching also for low VitD levels. VitD deficiency may accelerate pathogenesis and progression of CAD by enhancing metabolic impairment, inflammation and oxidative stress, that already characterize patients with MI and are even more significant in diabetic patients. In our study, increased CRP levels were associated with worse outcomes in the fully adjusted Cox regression model. We have previously demonstrated that VitD deficiency after MI is associated with adverse LV remodeling and, consequently, with higher HF and mortality rates [6,13]. However, despite the positive impact of VitD on cardiovascular health and the association of its deficiency with worse outcomes, several trials were not successful in demonstrating the therapeutic advantage of VitD supplementation, probably because of the difficulty to achieve adequate levels of 25(OH)D3. Moreover, a recent meta-analysis [60] including 21 randomized clinical trials did not support VitD supplementation for risk reduction of cardiovascular events, cardiovascular and all-cause mortality. We strongly believe that these negative results derive from the inverse J-curve between VitD levels and mortality [6,61]. The effect of VitD supplementation could be beneficial only with true deficiency, while previously used approaches only provided supplementation, without assessing VitD levels. Indeed, only in five clinical trials included in the meta-analysis patients had insufficient VitD levels, in seven trials baseline VitD levels were not available, while in nine trials patients had VitD levels within normal range or over 30 ng/mL [60]. Furthermore, since differences in age, body weight, polymorphisms in VitD binding protein and comorbidities may affect VitD absorption and bioavailability, we strongly believe that a therapeutic drug monitoring (TDM) approach should be used during administration. Therefore, it might be useful to attempt VitD supplementation to improve the outcome only in patients with hypovitaminosis D after MI, independently of presence of diabetes or not. It is well known that targeting the RAAS is the cornerstone of efforts to prevent or slow the LV remodeling progression after MI, given his critical role on its pathogenesis [16]. ACEIs/ARBs should be started as soon as possible within the first 24 h in order to maximize their benefits [62,63]. VitD optimization could be of high relevance as RAAS inhibitor, particularly when ACEIs/ARBs could not be initiated or up-titrated due to low blood pressure and hemodynamic disorders. Any benefit of the administration of VitD in the acute phase and during the follow-up as a RAAS inhibitor could be modest in patients with sufficient VitD levels. Therefore, a clinical trial adequately designed, using TDM approach for VitD supplementation is needed.

The major strength of our study is the large, well-defined and unselected population from single center and this makes possible for us to generalize its results to all patients after MI. Another strong point is the relatively lasting follow-up.

A possible objection could be that VitD was determined only once, during the index hospitalization for MI. However, in a longitudinal population-based cohort study, Jorde et al. [64] examined VitD levels over 14 years on the same subjects and demonstrated that measured levels of serum VitD tend to persist and that a significant improvement of VitD levels over time are improbable. Unfortunately, the observational design of our study excludes any conclusions about causality. Further, adequately designed trials on VitD optimization are necessary.

## 5. Conclusions

DM and insufficient VitD levels, individually and synergistically, are associated with a worse outcome in patients after an MI.

## Figures and Tables

**Table 1:** Clinical characteristics of the study population according to diabetes and Vitamin D status: group 1 with diabetes mellitus (DM) and hypovitaminosis D, group 2 with only hypovitaminosis D, group 3 with only DM, and group 4 patients without DM and hypovitaminosis D.

Variables	All Patients *n* = 1081	HypoVitD		No HypoVitD		
		DM Group 1 *n* = 255	No DM Group 2 *n* = 426	DM Group 3 *n* = 106	No DM Group 4 *n* = 294	*p* Value
Age *	66.7 (11.5)	64.6 (10.53)	67.7 (12.2)	66.6 (8.8)	62 (11.3)	0.016
Male gender (%) ˆ	70.9	72.9	66.9	83	70.4	0.010
BMI (kg/m^2^) †*•	26.9 (4.4)	28.2 (4.2)	26.5 (4.1)	27.3 (4.6)	26.2 (4)	<0.001
SBP (mmHg)	136.9 (25.2)	133.9 (24.8)	138.4 (23.9)	140.6 (29.8)	131.9 (20.1)	0.651
DBP (mmHg)	78.4 (13.9)	78.5 (14.6)	80.3 (14)	79.6 (14.1)	80.9 (11.5)	0.748
Heart rate (bpm)	75.6 (16.8)	76.4 (15.8)	75.4 (13.8)	83.4 (30.9)	76.2 (15)	0.308
LBBB (%)	4.4	5.1	4.9	5.7	2.7	0.404
Cardiac arrest (%)	2.7	2	2.2	1.9	4.1	0.369
Diagnosis: (%)						
STEMI	59.9	57.3	59.6	55.7	63.9	0.314
NSTEMI	40.1	42.7	40.4	44.3	36.1	
Killip > 1 (%) *	21.4	27.8	21.6	20.8	15.6	0.007
Killip Class (%) *						
I	78.6	72.2	78.4	79.2	84.4	0.071
II	15.4	18.4	16	14.2	12.6	
III	3.6	5.5	3.5	4.7	1.7	
IV	2.3	3.9	2.1	1.9	1.4	
Hypertension (%) †*	68.5	76.9	64.8	76.4	63.6	0.001
Dislipidemia (%) †*	56.9	64.3	54.3	65.1	51.4	0.004
Smoking (%)	38.9	38.4	34.9	36.4	38.9	0.354
Family history for IHD (%)	26.1	24.3	27.7	25.5	25.5	0.786
Peripheral artery disease (%) †*	9.1	18.8	5.9	11.3	4.4	<0.001
Previous MI/PTCA/CABG (%) *ˆ	18.8	22	17.4	32.1	13.3	<0.001
Hb (g/dL) †*	12.3 (11.2–13.6)	12.2 (11.1–13.6)	12.8 (11.4–14)	12.6 (11.6–14)	13 (12–14)	<0.001
Total cholesterol (mg/dL) †*ˆ•	183 (152.5–214.5)	185 (147–230)	193 (152.5–232)	165 (148–197.5)	193 (166.5–229.5)	<0.001
HDL cholesterol (mg/dL) †*ˆ•	43 (36–51)	39 (31–46)	44 (36–55)	43 (32.5–55)	46 (36.5–55.5)	<0.001
LDL cholesterol (mg/dL) †* ˆ•	113.6 (87.7–139.1)	118.6 (81.6–144.4)	119.2 (91.7–150)	90.6 (76.1–122.9)	124 (101.2–155.3)	<0.001
Triglycerides (mg/dL) †* ˆ•	110 (82–149.5)	132 (100–202)	111 (88–160.5)	136 (95–196)	110 (76.5–145.5)	<0.001
HbA1C% †*ˆ•	5.9 (5.5–6.5)	7.1 (6.5–8.9)	5.7 (5.6–6)	6.7 (6.1–7.9)	5.8 (5.6–6)	<0.001
Creatinine (mg/dL) †*	0.95 (0.79–1.2)	1 (0.8–1.2)	0.9 (0.8–1.1)	1 (0.8–1.3)	0.9 (0.8–1.1)	0.026
GFR mL/min/1.73 m2 per BSA †	71.4 (54.3–92.1)	68.4 (50.3–79.1)	62 (51–74.6)	61.1 (43.4–74.2)	68.8 (57.5–83.6)	0.081
GRACE score 6 months *	131.5 (34.4)	122.8 (29.2)	123.5 (29.5)	128.8 (32)	116.1 (34.3)	0.020
EDD_I (cm)	2.2 (1)	2.5 (0.5)	2.6 (0.4)	2.6 (0.3)	2.6 (0.3)	0.283
ESD_I (cm)	1.5 (0.8)	1.7 (0.5)	1.7 (0.4)	1.8 (0.4)	1.8 (0.4)	0.862
FS%	33.7 (10.9)	32.7 (12)	34.8 (9.1)	35 (13.7)	32.2 (10.5)	0.330
LVEF%	51.6 (11.3)	52 (10)	53 (9.9)	49.7 (10.6)	53.1 (8.01)	0.027
E/A	0.9 (0.7–1.2)	0.8 (0.7–1)	0.9 (0.7–1.2)	0.9 (0.7–1.1)	0.9 (0.7–1.2)	0.732
E/E’ratio †*§•	10.8 (9–14)	11.4 (9–16)	11 (9–13.8)	11 (9.5–187)	10 (8–11.1)	<0.001
WMSI	1.5 (0.4)	1.5 (0.4)	1.5 (0.4)	1.5 (0.4)	1.5 (0.3)	0.145
Mitral insufficiency: (%)						
Absent	37.8	36.3	38.5	36.7	38.4	0.801
Mild	54.8	55.9	54.3	54.1	54.8	
Moderate	6.4	6.1	6.9	8.2	5.3	
Severe	0.8	1.6	0.2	1	1.4	
PCI (%) *	66.2	59	69.1	64.2	68.9	0.034
CABG (%) ~*	13	20.7	9.7	11.3	11.6	<0.001
Medical therapy (%)	20.8	20.3	21.1	24.5	19.5	0.734
Time to PTCA (h)	3 (2–4.6)	3.1 (2.1–6.1)	2.5 (2–4.4)	2.4 (1.4–4.1)	3.1 (2–5.1)	0.440
Multivessel disease 70% (%) †*	38.4	47.4	36.5	46.2	30.7	<0.001
IIB/IIIA inhibitors (%)	13.4	12.1	12.2	14.9	15.9	0.552
NYHA class at discharge: (%) †ˆ						
I	83.1	71.8	87.1	77.1	89.3	< 0.001
II	14.4	24.6	10.1	22.9	9	
III	2.3	3.6	2.6	0	1.7	
IV	0.1	0	0.2	0	0	
Vitamin D (ng/mL) †~* ˆ§	16.7 (9.4–23.7)	10.8 (7.2–15)	13 (7.7–16.9)	24.7 (22.3–29.2)	26 (23.3–33.6)	<0.001
CRP (mg/L) †~*	22.7 (2.4–20.6)	7.4 (2.8–22)	6.3 (1.8–21.9)	5.8 (1.4–14.8)	3.9 (1.7–9.5)	<0.001
ACEIs/ARBs (%) †*	72.9	79.3	69.8	77.1	70.5	0.031
Beta blockers (%)	78.2	76.9	78.4	79	78.8	0.949
Amiodarone (%)	9.1	10.5	8.9	13.3	6.6	0.167
Antialdosteronics (%)	10.5	12.1	10.1	14.3	8.3	0.280
Loop diuretics (%) †*	23	33.2	21.3	25.7	15.6	<0.001
Aspirin (%)	93.6	97.4	93.5	89.5	94.1	0.313
P2Y12 inhibitors (%)	79.3	76.2	80.3	72.4	83	
Clopidogrel	52.6	52.2	56.1	45.7	50.2	0.062 0.048
Prasugrel	15.8	13	15.6	19	17.3	
Ticagrelor	14.7	13.8	12.9	12.4	19	
Statins (%) ˆ	87.4	87.4	85.9	94.3	87.2	0.142
Oral antidiabetics (%) †*ˆ	18.9	53.8	0	56.2	1.4	<0.001
Insulin (%) †*ˆ	8.8	24.3	0	29.5	0	<0.001
Warfarin (%)	7.9	10.9	6.2	12.4	5.9	0.026

† Statistical significance between group 1 vs. 2; ~ statistical significance between group 1 vs. 3; * statistical significance between group 1 vs. 4; ˆ statistical significance between group 2 vs. 3; § statistical significance between group 2 vs. 4; • statistical significance between group 3 vs. 4. Legend: BMI: body mass index; STEMI: ST-segment elevation myocardial; NSTEMI: non-ST-segment elevation myocardial infarction; IHD: ischemic heart disease; PTCA: percutaneous transluminal coronary angioplasty; CABG: coronary artery bypass grafting; HbA1C: hemoglobin A1c (glycated hemoglobin); CRP: C-reactive protein; Hb: hemoglobin; HF: heart failure; GFR: estimated glomerular filtration rate; EDD_I: end diastolic diameter indexed; ESD_I end systolic diameter indexed; IVS: interventricular septum; FS: fractional shortening; LBBB: left bundle branch block; LVEF: left ventricular ejection fraction; WMSI: wall motion score index; PCI: percutaneous coronary intervention; ACEIs/ARBs: angiotensin converting enzyme inhibitors/angiotensin receptor blockers; NYHA: New York Heart Association.

**Table 2:** Multivariable Cox proportional hazards regression analysis for primary composite end-point.

Variables	HR	95% CI	*p*
Age (for 1-year increase)	1.04	1.03–1.05	<0.0001
Previous MI/PCI/CABG	2.1	1.66–2.6	<0.0001
LVEF (for 10 point % increase)	0.83	0.75–0.91	<0.0001
CRP (quartiles)	1.18	1.07–1.3	0.001
ACEI/ARBs (yes vs. no)	0.79	0.63–1.005	0.055
DM (yes vs. no)	1.3	1.05–1.61	0.014
Vitamin D (below 20 ng/mL vs. over 20 ng/mL)	1.3	1.04–1.64	0.022

**Legend:** MI: myocardial infarction; PCI: percutaneous coronary intervention; CABG: coronary artery bypass grafting; LVEF: left ventricular ejection fraction; CRP: C-reactive protein; ACEIs/ARBs: angiotensin converting enzyme inhibitors/angiotensin receptor blockers; DM: diabetes mellitus.

## Supplementary Materials

The following figures and tables are available online at https://www.mdpi.com/2077-0383/9/7/2127/s1, Figure S1: Cumulative incidence of angina events taking into account death as a competing risk; Figure S2: Cumulative incidence of HF events taking into account death as a competing risk; Figure S3: Cumulative incidence of death; Table S1: Cumulative incidence rates of Angina/MI, taking into account death as a competing risk; Table S2: Cumulative incidence rates of HF, taking into account death as a competing risk.
