# Supplementary material for: Diabetes Mellitus and Vitamin D Deficiency: Comparable Effect on Survival and a Deadly Association after a Myocardial Infarction
Source: J Clin Med. 2020 Jul 6;9(7):2127. doi: 10.3390/jcm9072127 (PMC7408858; doi:10.3390/jcm9072127)
Supplement: Supplementary file 1 [file jcm-09-02127-s001.zip › jcm-856070-SI-conversion/Table S1.pdf]

Supplementary Table 1: Cumulative incidence rates of Angina/MI, taking into account death as a competing risk

Follow-up	12 months	24 months	48 months	96 months	120 months
**Groups of patients**		**Angina/MI (%)**			
DM & HypovitD	11.7	15.6	20.1	21.9	24.6
nonDM & HypovitD	8.7	13.8	16	19.9	22.7
DM & non HypovitD	10.3	11.6	13	18.2	23.6
Non DM & non HypovitD	6.7	11.1	14.6	16.7	16.7
		**Death before Angina/MI event (%)**			
DM & HypovitD	13.2	15.6	19.2	27.5	34.8
nonDM & HypovitD	6	8.9	13.9	21.2	24.8
DM & non HypovitD	5.1	7.5	9.7	24.8	30.4
Non DM & non HypovitD	5	6.4	7.6	17.4	21.6
