# Supplementary material for: Diabetes Mellitus and Vitamin D Deficiency: Comparable Effect on Survival and a Deadly Association after a Myocardial Infarction
Source: J Clin Med. 2020 Jul 6;9(7):2127. doi: 10.3390/jcm9072127 (PMC7408858; doi:10.3390/jcm9072127)
Supplement: Supplementary file 1 [file jcm-09-02127-s001.zip › jcm-856070-SI-conversion/Table S2.pdf]

Supplementary Table 2: Cumulative incidence rates of HF, taking into account death as a competing risk

Follow-up	12 months	24 months	48 months	96 months	120 months
**Groups of patients**		**HF (%)**			
DM & HypovitD	8.1	10.9	16.3	22	22.8
nonDM & HypovitD	6	7.8	9.8	12	12.6
DM & non HypovitD	6	12.1	13.6	16.2	16.2
Non DM & non HypovitD	4.4	5.3	7.1	8.9	9.9
		**Death before HF event (%)**			
DM & HypovitD	13,3	15,2	20	26,5	33,2
nonDM & HypovitD	5,2	9,1	13,2	22,7	25,6
DM & non HypovitD	6,2	7,4	7,4	19,7	28,8
Non DM & non HypovitD	5,4	6,4	7	15,5	24,3
