# Supplementary figures and images for: Diabetes Mellitus and Vitamin D Deficiency: Comparable Effect on Survival and a Deadly Association after a Myocardial Infarction
Source: J Clin Med. 2020 Jul 6;9(7):2127. doi: 10.3390/jcm9072127 (PMC7408858; doi:10.3390/jcm9072127)

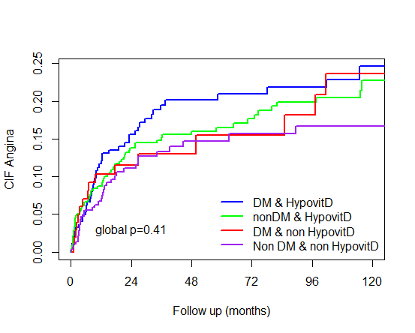

Supplement: Supplementary file 1 [file jcm-09-02127-s001.zip › jcm-856070-SI-conversion/Figure S1.jpg]

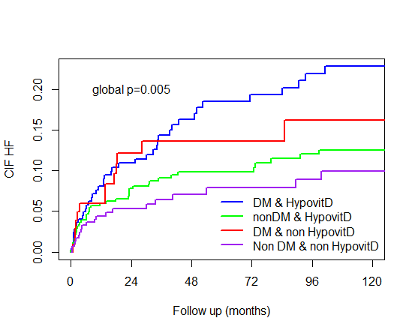

Supplement: Supplementary file 1 [file jcm-09-02127-s001.zip › jcm-856070-SI-conversion/Figure S2.jpg]

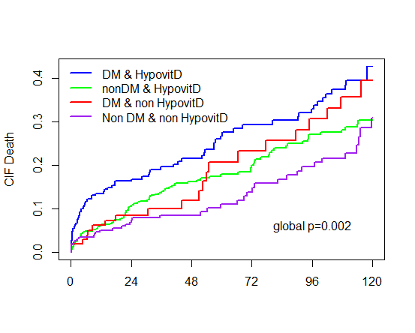

Supplement: Supplementary file 1 [file jcm-09-02127-s001.zip › jcm-856070-SI-conversion/Figure S3.jpg]
